# CCL4 Stimulates Cell Migration in Human Osteosarcoma via the mir-3927-3p/Integrin αvβ3 Axis

**DOI:** 10.3390/ijms222312737

**Published:** 2021-11-25

**Authors:** Hsiao-Chi Tsai, Yan-You Lai, Hsuan-Chih Hsu, Yi-Chin Fong, Ming-Yu Lien, Chih-Hsin Tang

**Affiliations:** 1 School of Medicine, China Medical University, Taichung 404022, Taiwan; moxa0110@gmail.com; 2 Division of Hematology and Oncology, Department of Internal Medicine, China Medical University Hospital, Taichung 404332, Taiwan; 3 School of Chinese Medicine, China Medical University, Taichung 406040, Taiwan; u110022305@cmu.edu.tw; 4 Department of Medical Research, Chung Shan Medical University, Taichung 40201, Taiwan; s1001141@gm.csmu.edu.tw; 5 Department of Sports Medicine, College of Health Care, China Medical University, Taichung 404022, Taiwan; yichin.fong@gmail.com; 6 Department of Orthopedic Surgery, China Medical University Beigang Hospital, Yunlin 651012, Taiwan; 7 Graduate Institute of Basic Medical Science, China Medical University, Taichung 404022, Taiwan; 8 Graduate Institute of Biomedical Sciences, China Medical University, Taichung 404022, Taiwan; 9 Chinese Medicine Research Center, China Medical University, Taichung 404022, Taiwan; 10 Department of Biotechnology, College of Health Science, Asia University, Taichung 41354, Taiwan

**Keywords:** CCL4, integrin, miR-3927-3p, metastasis, osteosarcoma

## Abstract

Osteosarcoma is the most common type of primary malignant bone cancer, and it is associated with high rates of pulmonary metastasis. Integrin αvβ3 is critical for osteosarcoma cell migratory and invasive abilities. Chemokine (C-C motif) ligand 4 (CCL4) has diverse effects on different cancer cells through its interaction with its specific receptor, C-C chemokine receptor type 5 (CCR5). Analysis of mRNA expression in human osteosarcoma tissue identified upregulated levels of CCL4, integrin αv and β3 expression. Similarly, an analysis of records from the Gene Expression Omnibus (GEO) dataset showed that CCL4 was upregulated in human osteosarcoma tissue. Importantly, the expression of both CCL4 and integrin αvβ3 correlated positively with osteosarcoma clinical stages and lung metastasis. Analysis of osteosarcoma cell lines identified that CCL4 promotes integrin αvβ3 expression and cell migration by activating the focal adhesion kinase (FAK), protein kinase B (AKT), and hypoxia inducible factor 1 subunit alpha (HIF-1α) signaling pathways, which can downregulate microRNA-3927-3p expression. Pharmacological inhibition of CCR5 by maraviroc (MVC) prevented increases in integrin αvβ3 expression and cell migration. This study is the first to implicate CCL4 as a potential target in the treatment of metastatic osteosarcoma.

## 1. Introduction

Osteosarcoma, the most common primary bone malignancy, is renowned for its high propensity for metastasis [1]. As many as 20% of patients present with lung metastases at their initial diagnosis; around 40% of patients develop metastases after their initial presentation despite the best available treatment [2]. Whereas 5-year overall survival is around 50–80% in patients with localized osteosarcoma [1], only 27% of patients with metastatic disease survive longer than 5 years [3]. No agents exist that can successfully cure metastatic osteosarcoma, so it is essential that such treatment is developed [4].

The transformation of normal cells into cancer cells is a complex process, in which integrins play a critical role by mediating cell-to-cell adhesion and their adhesion to the extracellular matrix [5]. Notably, higher levels of integrin expression enhance metastasis in different types of cancers [6], including osteosarcoma [7,8,9]. Some research has suggested that integrin may be a promising therapeutic target for osteosarcoma [10]. Integrin αvβ3 is involved in cell migration and induces osteosarcoma metastasis [11]. Analysis of integrin αvβ3 expression in primary osteosarcoma specimens has been found to be associated with poor clinical outcomes and correlated with shorter disease-free survival and overall survival [12]. Thus, integrin αvβ3 may represent an attractive therapeutic target for the treatment of osteosarcoma [12]. Moreover, it is established that microRNAs (miRNAs, miRs), small endogenous RNAs that regulate multiple biological functions of osteosarcoma and mediate cancer cell interactions with the extracellular matrix, can inhibit the disease process of osteosarcoma. For example, the downregulation of integrin-linked kinase (ILK) and integrin α6 by miR-542-3p and miR-127-3p, respectively, inhibits osteosarcoma cell proliferation, migration and invasion [13,14,15]. Overexpression of miR-548c-3p inhibits levels of integrin by directly targeting the 3’-untranslated region (3′UTR) of osteosarcoma cells [16].

Another critically important player in the tumor microenvironment is chemokine C-C motif ligand 4 (CCL4), which can both facilitate protumorigenic capacities and also enhance tumor immunity, depending on its interactions with C-C chemokine receptor type 5 (CCR5) [17]. For instance, upregulated CCL4 levels exhibit protumorigenic activities in many different types of cancer [18]. In endometrial and oral cancers, CCL4 reportedly causes angiogenesis and lymphangiogenesis by upregulating VEGF-A and VEGF-C expression, and facilitates metastasis [17,19,20,21]. CCL4 has also been found to inhibit normal osteoblast function, increase osteoclast activity and stimulate bone destruction [18], but its effect in osteosarcoma is unclear. This study is the first to investigate whether CCL4 has any direct effect in osteosarcoma. It was found that CCL4 affects integrin αvβ3 expression and osteosarcoma cell migration, so the study sought to determine which signaling pathways underlie these activities.

## 2. Results

### 2.1. Clinicopathological Characteristics of CCL4 in Osteosarcoma Tissue

**Figure 1:**
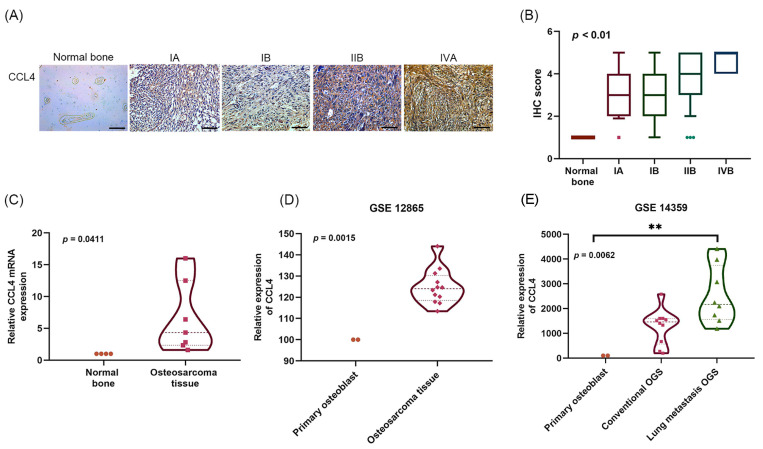
Clinicopathological characteristics of CCL4 in human osteosarcoma tissue. (**A**,**B**) Normal bone and osteosarcoma specimens were subjected to immunohistochemistry (IHC) evaluations with anti-CCL4 monoclonal antibody (scale bar = 100 μm). Staining intensity was graded as 0 (no staining), 1 (<25% of the cells with positive staining), 2 (25–49% of the cells with positive staining), 3 (50–74% of the cells with positive staining), or 4 (50–100% of the cells with positive staining). (**C**) CCL4 mRNA expression in normal bone and osteosarcoma tissue in the study hospital samples was detected by qPCR. (**D**) CCL4 mRNA expression in primary osteoblast and osteosarcoma tissue was analyzed using records from the Gene Expression Omnibus (GEO) Dataset GSE12865. (**E**) CCL4 mRNA expression in primary osteoblasts, conventional osteosarcoma (OGS), and lung metastatic OGS was analyzed using records from the GEO Dataset GSE14359. ** *p* < 0.01 compared with the control group.

IHC staining and qPCR were performed to detect levels of CCL4 in osteosarcoma tissue. Levels of CCL4 expression were higher in osteosarcoma tissue than in normal bone tissue (Figure 1A–C), and significant associations were observed with clinical disease stages (Figure 1A,B). Analysis of GEO Dataset osteosarcoma tissue samples revealed increased levels of CCL4 compared with levels in primary osteoblasts (Figure 1D), especially in lung metastatic osteosarcoma tissue (Figure 1E). These findings suggest that CCL4 is overexpressed in osteosarcoma and is associated with lung metastasis.

### 2.2. A Positive Correlation between CCL4 and Integrin αvβ3 Expression in Osteosarcoma Tissue

**Figure 2:**
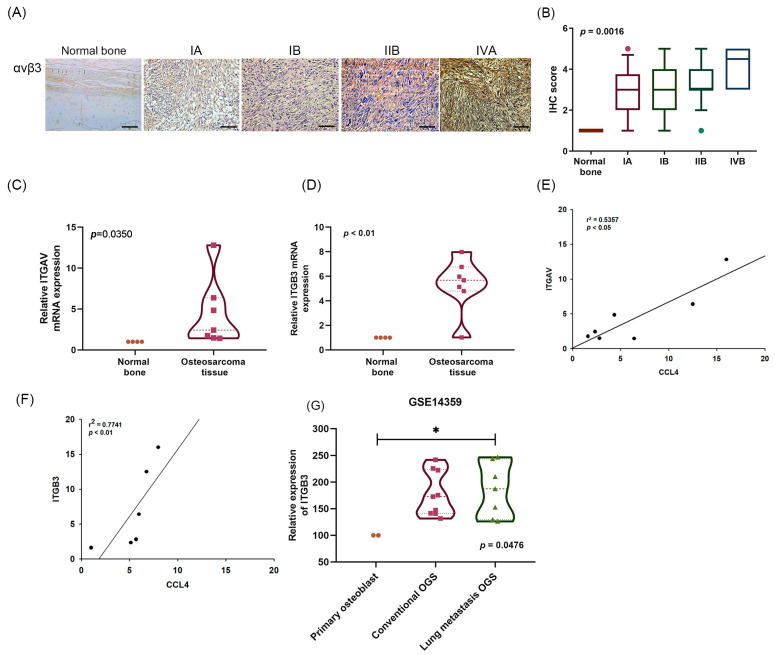
A positive correlation between CCL4 and integrin αvβ3 expression in osteosarcoma tissue. (**A**,**B**) Normal bone and osteosarcoma specimens were subjected to IHC evaluations with anti-integrin αvβ3 monoclonal antibody and the staining intensity was graded as 0 (no staining), 1 (<25% of the cells with positive staining), 2 (25–49% of the cells with positive staining), 3 (50–74% of the cells with positive staining), or 4 (50–100% of the cells with positive staining) (scale bar = 100 μm). (**C**,**D**) Levels of integrin αv (ITGAV) (**C**) and integrin β3 (ITGB3) (**D**) mRNA expression in normal bone and osteosarcoma tissue were detected by qPCR. Analysis of correlations between CCL4 and ITGAV (**E**) or ITGB3 (**F**) mRNA expression in osteosarcoma tissue. (**G**) Levels of integrin β3 (ITGB3) mRNA expression in primary osteoblasts, conventional OGS, and lung metastatic OGS were analyzed using records from the GEO Dataset GSE14359. * *p* < 0.05 compared with controls.

Abnormal expression of integrin αvβ3 in human osteosarcoma cells has been associated with cell motility and tumor progression [7,11]. Similarly, markedly higher expression of integrin αv and β3 was found in osteosarcoma tissue samples than in normal bone tissue (Figure 2A–D), and this higher expression was positively correlated with the clinical stages of disease (Figure 2A,B). Significant positive correlations were found between CCL4 expression and integrin αv and β3 in clinical osteosarcoma tissues (Figure 2E,F). The GEO database analysis revealed significantly higher integrin β3 expression in lung metastatic osteosarcoma tissue (Figure 2G). These data demonstrate that the expression of both CCL4 and integrin αvβ3 correlate positively with osteosarcoma clinical stages and lung metastasis.

### 2.3. CCL4 Promotes Integrin αvβ3 Expression and Cell Migration through CCR5

**Figure 3:**
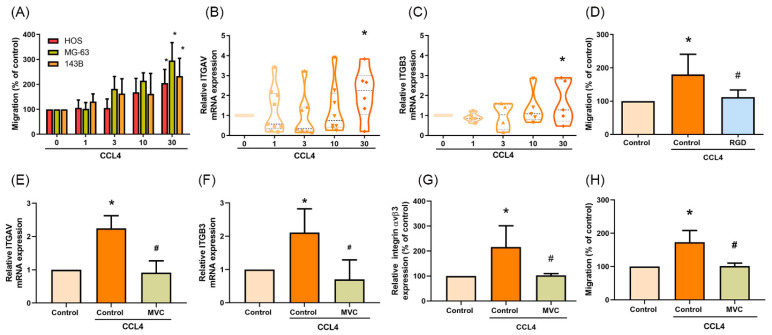
CCL4 promotes integrin αvβ3 expression and cell migration through the CCR5 receptor. (**A**) Cells were incubated with different concentrations of CCL4 (0–30 ng/mL) for 24 h, then cell migration ability was examined by the Transwell assay. (**B**,**C**) 143B cells were incubated with different concentrations of CCL4 (0–30 ng/mL) for 24 h, then mRNA expression of integrin αv (ITGAV) and β3 (ITGB3) was examined by qPCR. (**D**) 143B cells were treated with specific αvβ3-blocking peptide (RGD; 100 nM) for 30 min and then stimulated with CCL4 (30 ng/mL) for 24 h. Cell migration ability was examined by the Transwell assay. (**E**–**H**) 143B cells were treated with a CCR5 antagonist (maraviroc, MVC; 5 μM) for 30 min then stimulated with CCL4 (30 ng/mL) for 24 h. Levels of ITGAV (**E**) and ITGB3 (**F**) mRNA expression, cell surface integrin αvβ3 protein expression (**G**), as well as cell migratory abilities (**H**) were examined by qPCR, flow cytometry, and the Transwell assay, respectively. The results were obtained from three independent experiments and are expressed as the means ± SD. * *p* < 0.05 compared with controls; ^#^ *p* < 0.05 compared with CCL4-treated controls.

When the osteosarcoma cell lines were treated with different concentrations of CCL4, cell migration ability was increased 2-fold (Figure 3A and Supplementary Materials Figure S1). Moreover, mRNA expression of both integrin αv and β3 was upregulated after CCL4 treatment (Figure 3B,C) and this activity stopped when cells were pretreated with the specific αvβ3-blocking peptide (RGD) (Figure 3D and Supplementary Materials Figure S2). These results indicate that CCL4 enhances cell migration via integrin αvβ3. Disrupting CCL4-CCR5 signaling by the CCR5 antagonist (maraviroc, MVC) potently inhibited CCL4-induced integrin αv and β3 mRNA, cell surface integrin αvβ3 protein expression (Figure 3E–G), as well as cell migration abilities (Figure 3H and Supplementary Materials Figure S2). Thus, CCR5 plays an important role in the migration and levels of integrin αvβ3 expression in human osteosarcoma cells.

### 2.4. CCL4 Promotes Cell Migration by Activating FAK and AKT Signaling

**Figure 4:**
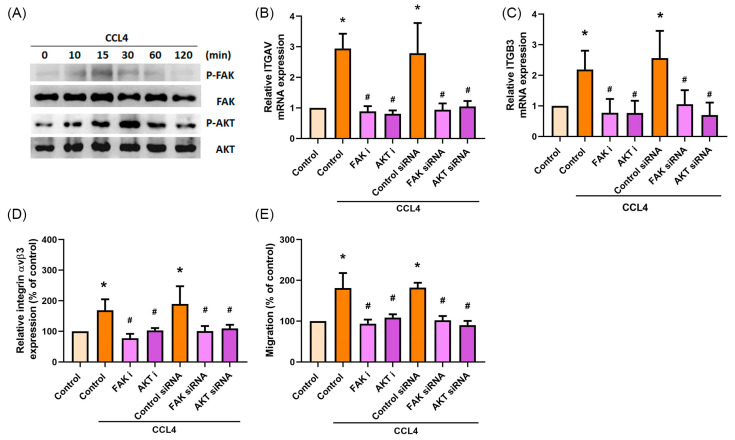
CCL4 promotes cell migration by activating FAK and AKT signaling. (**A**) 143B cells were incubated with CCL4 (30 ng/mL) for the indicated time intervals, then FAK and AKT activation was examined by the Western blot assay. (**B**–**E**) 143B cells were treated with a FAK inhibitor (FAK i, 10 μM) or AKT inhibitor (AKT i, 10 μM) for 30 min, or transfected with FAK or AKT siRNAs, then stimulated with CCL4 (30 ng/mL) for 24 h. Levels of ITGAV mRNA (**B**) and ITGB3 mRNA expression (**C**), cell surface integrin αvβ3 protein expression (**D**), and cell migratory abilities (**E**) were examined by qPCR, flow cytometry, and the Transwell assay, respectively. The results were obtained from three independent experiments and are expressed as the means ± SD. * *p* < 0.05 compared with controls; ^#^ *p* < 0.05 compared with CCL4-treated controls.

FAK and AKT signaling play a critical role in cancer cell adhesion, migration, and invasion [22,23,24]. This study therefore examined whether FAK and AKT signaling is involved in CCL4-mediated osteosarcoma cell migration. After incubating the cells with CCL4, levels of FAK and AKT phosphorylation were increased after 15 and 30 min, respectively (Figure 4A), but not after 24 h (Supplementary Materials Figure S3). Pretreating cells with FAK or AKT inhibitors and their respective siRNAs eliminated CCL4-induced integrin αv and β3 mRNA expression (Figure 4B,C). Similarly, the blockade of FAK and AKT signaling suppressed integrin αvβ3 expression and cell migration (Figure 4D,E and Supplementary Materials Figure S2). Furthermore, p-AKT was inhibited by the FAK inhibitor (Supplementary Materials Figure S4). Thus, AKT is a downstream molecule of FAK. CCL4 regulates integrin αvβ3 expression and migratory activities in osteosarcoma cells via the FAK/AKT signaling pathway.

### 2.5. HIF-1α Is Involved in CCL4-Mediated Expression of Integrin αvβ3 and Migratory Activities of Osteosarcoma Cells

**Figure 5:**
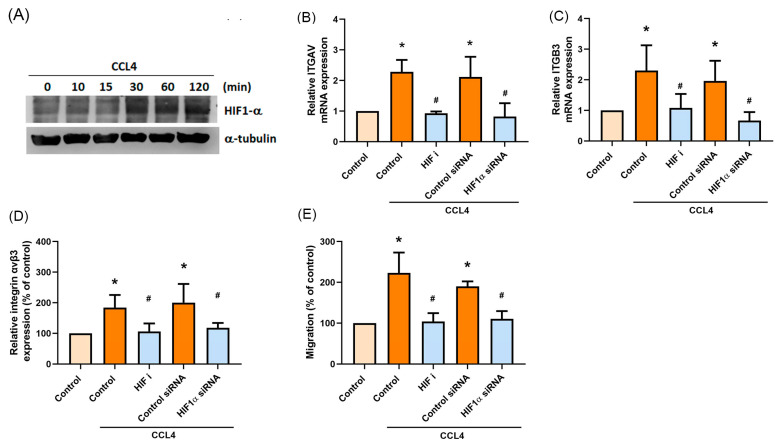
HIF-1α is involved in CCL4-mediated expression of integrin αvβ3 expression and migratory activities of osteosarcoma cells. (**A**) 143B cells were incubated with CCL4 (30 ng/mL) for the indicated time intervals, then HIF-1α activation was examined by the Western blot assay. (**B**–**E**) 143B cells were treated with an HIF-1α inhibitor (HIF i; 10 μM) for 30 min, or transfected with HIF-1α siRNA, then stimulated with CCL4 (30 ng/mL) for 24 h. Levels of ITGAV mRNA (**B**) and ITGB3 mRNA expression (**C**), cell surface integrin αvβ3 protein expression (**D**), and cell migratory abilities (**E**) were examined by qPCR, flow cytometry, and the Transwell assay, respectively. The results were obtained from three independent experiments and are expressed as the means ± SD. * *p* < 0.05 compared with controls; ^#^ *p* < 0.05 compared with CCL4-treated controls.

Tumor hypoxia is a common feature in most solid tumors and is associated with metastasis [25,26]. To analyze the mechanisms underlying CCL4-mediated migration of osteosarcoma cells, the Western blot assay examined HIF-1α expression. Treating cells with different concentrations of CCL4 increased HIF-1α protein expression (Figure 5A). Inhibiting HIF-1α expression significantly decreased levels of integrin αvβ3 mRNA and protein expression (Figure 5B–D) and also cell migratory activity (Figure 5E and Supplementary Materials Figure S2). Furthermore, HIF-1α was inhibited by the FAK and AKT inhibitors (Supplementary Materials Figure S4). These results indicate that CCL4-induced integrin αvβ3 expression and stimulation of osteosarcoma cell migration occurs through the FAK/AKT/HIF-1α signaling pathway.

### 2.6. Downregulation of miR-3927-3p Increases Levels of Integrin αvβ3 Expression and Osteosarcoma Cell Migration

**Figure 6:**
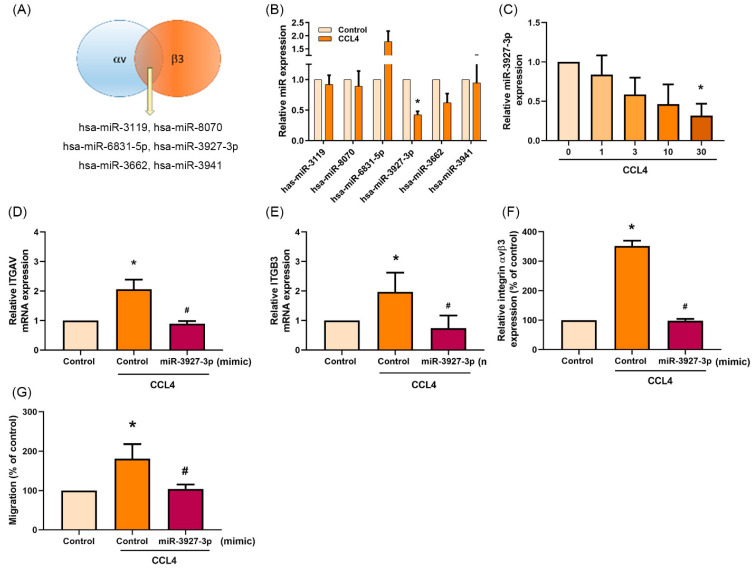
Downregulation of miR-3927-3p increases levels of integrin αvβ3 expression and osteosarcoma cell migration. (**A**) The miRDB database (http://mirdb.org/miRDB, accessed on 19 June 2019) was used to predict miRNAs that potentially bind to the integrin αv- and β3-3′UTRs. (**B**) 143B cells were incubated with CCL4 (30 ng/mL) for 24 h and the qPCR assay determined levels of expression for all indicated miRNAs. (**C**) 143B cells were incubated with CCL4 (0–30 ng/mL) for 24 h and miR-3927-3p expression was determined by qPCR. (**D**–**G**) 143B cells were transfected with miR-3927-3p mimic then stimulated with CCL4 for 24 h. Levels of ITGAV mRNA (**D**), ITGB3 mRNA (**E**), cell surface integrin αvβ3 protein expression (**F**), and cell migratory abilities (**G**) were examined by qPCR, flow cytometry, and the Transwell assay, respectively. The results were obtained from three independent experiments and are expressed as the means ± SD. * *p* < 0.05 compared with controls; ^#^ *p* < 0.05 compared with CCL4-treated controls.

An online open source database for miRNA target prediction and functional annotations, miRDB, identified six miRNAs that may directly interfere with both integrin αv and β3 (Figure 6A). The expression of all six miRNAs was detected in osteosarcoma cells after CCL4 treatment. The expression of miR-3927-3p was significantly downregulated (Figure 6B,C). After transfecting osteosarcoma cells with miR-3927-3p mimic, mRNA expression of integrin αv and β3 was inhibited (Figure 6D–F), as was cell migratory activity (Figure 6G and Supplementary Materials Figure S2).

**Figure 7:**
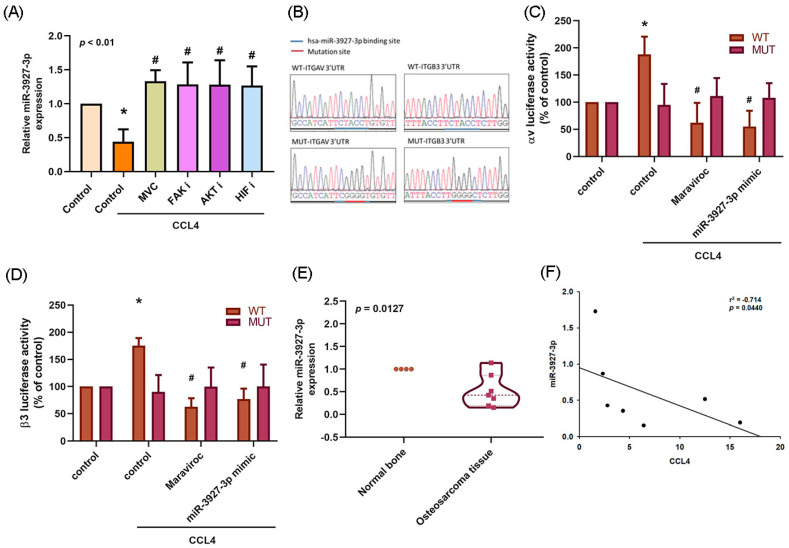
The FAK/AKT/HIF-1α signaling pathway is involved in CCL4-downregulated miR-3927-3p expression. (**A**) 143B cells were treated for 30 min with a FAK i (10 μM), AKT i (10 μM), or HIF i (10 μM) then incubated with CCL4 for 24 h. miR-3927-3p expression was examined by qPCR. (**B**) The wild-type (WT) or mutant binding sites (MUT) of ITGAV and ITGB3 3′UTRs containing the miR-3927-3p binding site were inserted into the pmirGLO vector. (**C**,**D**) The WT- or MUT-3′UTR luciferase activities of integrin αv and β3 were measured after MVC treatment or transfection with miR-3927-3p mimic. (**E**) miR-3927-3p expression in normal bone and osteosarcoma tissues were detected by qPCR. (**F**) Analysis of the correlation between CCL4 and miR-3927-3p expression in osteosarcoma tissue. The results were obtained from three independent experiments and are expressed as the means ± SD. * *p* < 0.05 compared with controls; ^#^ *p* < 0.05 compared with CCL4-treated controls.

Treating osteosarcoma cells with CCR5, FAK, AKT or HIF-1α inhibitors reversed CCL4-mediated inhibition of miR-3927-3p expression, which suggests that CCL4 may increase integrin αvβ3 expression and cell migration by inhibiting miR-3927-3p synthesis via CCR5 and FAK/AKT/HIF-1α signaling (Figure 7A). CCL4 increased the WT but not MUT binding sites of the 3′UTR luciferase plasmids, which confirmed that miR-3927-3p directly binds to the 3′UTR of integrin αv and β3 (Figure 7B,C). Further analyses revealed that miR-3927-3p expression was downregulated in osteosarcoma tissues (Figure 7D) and was negatively correlated with CCL4 expression (Figure 7E). Thus, miR-3927-3p suppresses cell migration by binding to the 3′UTR of the human integrin αv and β3 gene via CCR5 and FAK/AKT/HIF-1α signaling. MiR-3927-3p plays a critical role in CCL4-mediated integrin αvβ3 expression and cell migration.

## 3. Discussion

Osteosarcoma is associated with high rates of pulmonary metastasis [1]. The prognosis for patients with pulmonary metastasis is worse than for those with localized disease, characterized by substantially reduced survival [3]. Understanding the molecular mechanisms of metastatic osteosarcoma is essential for developing therapeutic interventions for metastatic osteosarcoma. In this study, our investigation into whether CCL4 affects osteosarcoma cell migration found that CCL4 promotes integrin αvβ3 expression by downregulating miR-3927-3p through the CCR5 and FAK/AKT/HIF-1α signaling pathway. These data provide new insights for metastatic osteosarcoma in the clinic.

CCR5 is important in the development of many different types of cancers, including prostate, colon, breast, ovarian and cervical cancers [27]. Inhibiting the interaction of CCR5 and its ligands underlies the clinical application of various anticancer agents [28]. The CCR5 antagonists TAK-779, anibamine and maraviroc (MVC) have proven to be effective in the treatment of various cancers [29]. The combination of MVC and pembrolizumab was used to treat 20 patients with refractory microsatellite stable (MSS) metastatic colorectal cancer in a phase I trial (ClinicalTrials.gov Identifier: NCT03274804), while in a phase II clinical trial, the efficacy of MVC was examined in the prophylaxis of graft-versus-host disease (GVHD) in patients with hematologic malignancies (NCT01785810). Results from the phase I trial supported the feasibility of combined MVC and pembrolizumab treatment and good toxicity, with higher-than-expected overall survival [30]. The results of the phase II trial are awaited. In another phase I study (NCT01736813) involving 12 patients with advanced colorectal cancer and hepatic liver metastases (all of whom were treatment-refractory to standard-of-care therapies), MVC treatment dramatically ameliorated the tumor-promoting environment and showed high response rates [31]. Here, our use of MVC to block the function of the CCL4/CCR5 axis successfully inhibited integrin αvβ3 expression as well as the migration of osteosarcoma cells, indicating that MVC has therapeutic efficacy in osteosarcoma metastasis.

As HIF pathways have proven to be relevant in tumor pathogenesis and miRNAs play pivotal roles in gene expression, many research groups have explored the transcriptional output of miRNAs in HIF-associated malignant progression [32]. In pancreatic cancer, HIF-1α recruits histone deacetylase 1 (HDAC1) to the promoter of miR-548an, which transcriptionally suppresses miR-548an expression and subsequently upregulates vimentin, which facilitates pancreatic tumorigenesis [33]. Experimental evidence from research into a hepatocellular carcinoma cell line has shown that HIF-1α mediates vasodilator-stimulated phosphoprotein (VASP) overexpression at the transcriptional level by directly binding to the promoter of the VASP gene [34]. Furthermore, HIF-1α can also inhibit miR-204 and thereby upregulate VASP at the post-transcriptional level [34]. Thus, HIF-1α can both directly and indirectly mediate the same gene in different ways [34]. HIF-1α is also capable of indirectly interfering with the tumor-suppressive ribonuclease III enzyme Dicer, a key player in the biogenesis of miRNAs [32,35]. Under hypoxic stress, epidermal growth factor receptor (EGFR) suppresses the maturation of tumor-suppressor-like miRNAs by phosphorylating protein argonaute-2 and subsequently reduces the binding of Dicer to protein argonaute-2, inhibiting miRNA processing from precursor miRNAs to mature miRNAs [36]. In this study, we found that miR-3927-3p was downregulated by the FAK/AKT/HIF-1α signaling pathway. Whether this signaling cascade directly or indirectly regulates miR-3927-3p expression via HIF-1α needs further investigation.

Experimental and clinical evidence has shown that integrin is necessary for cancer cell biological functions and that it promotes certain activities critical for tumor progression and metastasis [37,38], including inflammatory cell recruitment, extracellular matrix remodeling and tumor angiogenesis [37]. The expression of integrin αvβ3, a5β1 and αvβ6 is low or even undetectable in most adult epithelia, but often greatly upregulated in tumors [39]. Targeting integrin αvβ3 has shown promising antitumor activity [40]. High expression of integrin αvβ3 in patients with osteosarcoma is significantly associated with a poor response to chemotherapy as well as poor disease-free and overall survival [12]. Integrin αvβ3 induces migratory, invasive and antiapoptotic activities of osteosarcoma cells [41]. Disappointingly, the therapeutic expectations from preclinical trials have failed to translate into clinical studies employing strategies that inhibit integrin αvβ3 [37]. For instance, the development of the integrin antagonist cilengitide was halted when a phase III trial failed to show that the addition of cilengitide to temozolomide chemoradiotherapy was of any benefit in patients with newly diagnosed glioblastoma and MGMT promoter methylation [42]. Moreover, other clinical trials involving integrin αvβ5 and α5β1 inhibitors have failed to demonstrate therapeutic benefits, and no integrin inhibitors have been registered as anticancer drugs [37]. Perhaps an incomplete understanding of integrin function and biology has meant that researchers have failed to fully appreciate the pharmacokinetic parameters, or to understand the complexity involving intrinsic properties of integrins. Failing to recognize these shortcomings would feasibly lead to the failure of preclinical drugs to have any discernable impact upon integrins. Notably, these gaps in understanding should stimulate scientists to think about developing new concepts, tools and approaches to successfully exploit these molecules [37]. Instead, targeting the upstream expression of integrin subunits may prove to be worthwhile.

## 4. Materials and Methods

### 4.1. Materials

Human recombinant CCL4 protein was purchased from PeproTech (Rocky Hill, NJ, USA). Antibodies used in this study, phospho-focal adhesion kinase (p-FAK) and phospho-protein kinase B (p-AKT), were purchased from Cell Signaling Technology (Danvers, MA, USA); antibodies specific for FAK, AKT, α-tubulin and integrin αv/β3 were purchased from Santa Cruz Biotechnology (Santa Cruz, CA, USA); hypoxia-inducible factor 1-alpha (HIF-1α) was purchased from Cambridge, UK (see Supplementary Materials Table S1 for details about the antibodies used in the study). Small interfering RNAs (siRNAs) against FAK and AKT were purchased from Dharmacon Research (Lafayette, CO, USA). The HIF1-α siRNA was purchased from Abcam (Cambridge, MA, USA). FAK, AKT, and HIF1-α inhibitors were purchased from Calbiochem (San Diego, CA, USA). Lipofectamine 2000 and miR-3927-3p mimic were purchased from Invitrogen (Carlsbad, CA, USA). Reporter lysis buffer was purchased from Promega (Madison, WI, USA). All other chemicals were purchased from Sigma-Aldrich (St. Louis, MO, USA).

Osteosarcoma tissue samples were collected from 7 patients undergoing surgical resection in China Medical University Hospital. Written informed consent was obtained from each study participant before enrollment. This study was approved by the Institutional Review Board of China Medical University Hospital, and it was conducted according to the Declaration of Helsinki guidelines. Commercial osteosarcoma tissue arrays (T261 and OS804d) were purchased from US Biomax, Inc. (Rockville, MD, USA).

### 4.2. Cell Culture

Human osteosarcoma cell lines MG-64, HOS and 143B were purchased from the Bioresource Collection and Research Center (BCRC) (Hsinchu, Taiwan). Culture conditions of osteosarcoma cells followed those described in our previous studies [43,44].

### 4.3. Immunohistochemistry

The osteosarcoma tissue slides were baked for 120 min at 60 °C, then deparaffinized with xylene. The staining was performed by NovoLink Polymer System (Leica Microsystems), according to previously described methodology [45]. Immunostaining intensity of CCL4 and integrin αvβ3 was scored by MacBiophotonics ImageJ software (version 1.45). Positive staining intensity was graded as 0 (no positivity), (1) (1–24% positive), (2) (25–49% positive), (3) (50–74% positive), or (4) (50–100% positive).

### 4.4. Analysis of Messenger RNA (mRNA) Expression Profiles from the GEO Database

Screening of the Gene Expression Omnibus (GEO) Datasets revealed two microarrays related to osteosarcoma: GSE12865 and GSE14359. The GSE12865 profile is a genome-wide comparison of gene expression and consists of genes that are differentially expressed in 12 osteosarcoma tumor samples relative to 2 normal human osteoblast samples. The GSE14359 dataset contains 10 frozen conventional osteosarcoma and 8 lung metastatic osteosarcoma samples, as well as 2 normal human osteoblast samples.

### 4.5. Migration Assay

A sterile 6.5 mm Transwell kit with 8.0 µm-pore polycarbonate membrane inserts (Corning, NY, USA) was used for the cell migration assay, according to the established protocol [46]. Cell migration was quantified by counting the number of stained cells under a microscope.

### 4.6. Quantitative Real-Time PCR

Total RNA was extracted from osteosarcoma cells using TRIzol reagent. Total RNA (2 μg) was reverse-transcribed (RT) into complementary DNA (cDNA) using an oligo(dT) primer. The Mir-X™ miRNA First-Strand Synthesis was used to detect miRNA expression. The specific primer sequence for miR-3927-3p was 5′-CAGGTAGATATTTGATAGGCAT-3′. The qPCR assay was performed using the StepOnePlus sequence detection system, according to the established protocol [47,48,49]. Levels of GAPDH or U6 snRNA expression served as the endogenous control for normalization purposes.

### 4.7. Flow Cytometry

Osteosarcoma cells were harvested using 0.05% trypsin and then fixed in 2% formaldehyde, before incubating the mixture for 10 min at 4 °C. The cells were stained with anti-integrin αvβ3, then analyzed by fluorescent-activated cell sorting (FACS) on a FACScan flow cytometer (Becton Dickinson).

### 4.8. Western Blot Analysis

Cell lysates were prepared by RIPA buffer containing a protease inhibitor cocktail, then the protein was resolved with SDS-PAGE and transferred to Immobilon PVDF membranes. The protein was analyzed by Western blot, according to previously detailed procedures [50].

### 4.9. Luciferase Reporter Assay

The wild-type (WT) and mutant binding sites (MUT) of integrin αv- and β3-three prime untranslated region (3′UTR) luciferase plasmids were constructed by MDBio, Inc. (Taipei, Taiwan). The miR-3927-3p-binding regions of integrin αv and β3 were identified by the miRDB database (http://mirdb.org/miRDB, accessed on 21 June 2019). Cells were transfected with luciferase plasmids using Lipofectamine 2000 (Invitrogen, MA, USA), then treated for an additional 24 h with CCL4. Luciferase activity was calculated by the Luciferase Reporter Assay System (Promega, WI, USA), according to the manufacturer’s protocol.

### 4.10. Statistics

All statistical data were analyzed by GraphPad Prism 8.0 (GraphPad Software, CA, USA) and are expressed as the mean ± standard deviation (S.D.). Statistical comparisons between two samples were performed using the Student’s *t*-test. One-way analysis of variance (ANOVA) with post hoc Bonferroni correction was conducted for statistical analyses of multiple groups. In all cases, a *p*-value of <0.05 was considered significant.

## 5. Conclusions

**Figure 8:**
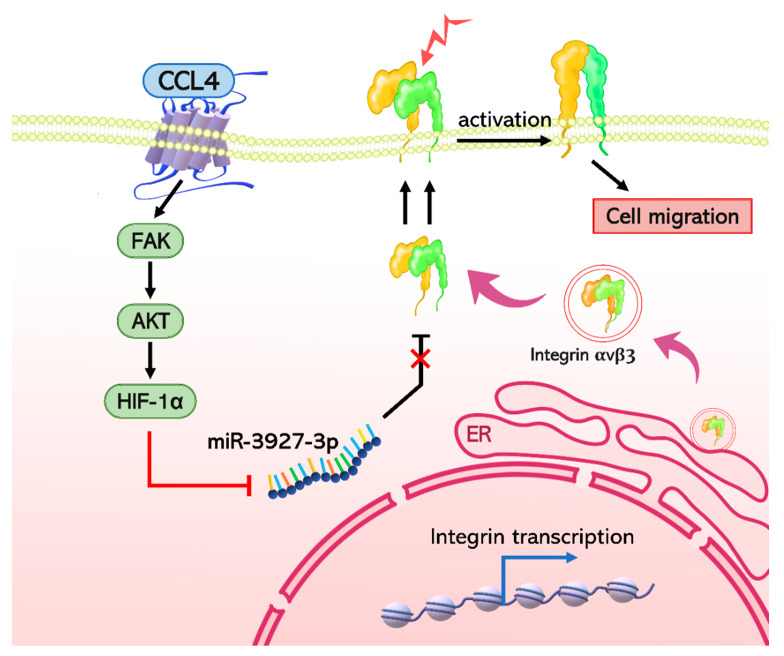
The schema depicts the involvement of signaling pathways in CCL4-induced stimulation of integrin αvβ3 expression and cell migration.

This study found high levels of CCL4 expression in clinical osteosarcoma tissue samples that correlated with clinical disease stages as well as the lung metastatic properties of this disease. According to the evidence, CCL4 promotes integrin αvβ3 expression through the CCR5 receptor and then activates the FAK/AKT/HIF-1α signaling pathway, which subsequently inhibits miR-3927-3p expression and promotes integrin αvβ3 expression, as well as osteosarcoma cell migration (Figure 8). CCL4 appears to be a new molecular therapeutic target in osteosarcoma metastasis.

## Data Availability

The raw data supporting the conclusions of this study are not available.

## Supplementary Materials

The following are available online at https://www.mdpi.com/article/10.3390/ijms222312737/s1.

## References

[1] Norregaard K.S., Jurgensen H.J., Gardsvoll H., Engelholm L.H., Behrendt N., Soe K. (2021). Osteosarcoma and Metastasis Associated Bone Degradation-A Tale of Osteoclast and Malignant Cell Cooperativity. Int. J. Mol. Sci..

[2] Taran S.J., Taran R., Malipatil N.B. (2017). Pediatric Osteosarcoma: An Updated Review. Indian J. Med. Paediatr. Oncol..

[3] Yu X., Yustein J.T., Xu J. (2021). Research models and mesenchymal/epithelial plasticity of osteosarcoma. Cell Biosci..

[4] Zhao X., Wu Q., Gong X., Liu J., Ma Y. (2021). Osteosarcoma: A review of current and future therapeutic approaches. Biomed. Eng. Online.

[5] Li M., Wang Y., Li M., Wu X., Setrerrahmane S., Xu H. (2021). Integrins as attractive targets for cancer therapeutics. Acta Pharm. Sin. B.

[6] Seguin L., Desgrosellier J.S., Weis S.M., Cheresh D.A. (2015). Integrins and cancer: Regulators of cancer stemness, metastasis, and drug resistance. Trends Cell Biol..

[7] Liu J.F., Chen P.C., Chang T.M., Hou C.H. (2020). Thrombospondin-2 stimulates MMP-9 production and promotes osteosarcoma metastasis via the PLC, PKC, c-Src and NF-kappaB activation. J. Cell Mol. Med..

[8] Jiang Y., Luo Y. (2020). LINC01354 Promotes Osteosarcoma Cell Invasion by Up-regulating Integrin beta1. Arch. Med. Res..

[9] Li R., Shi Y., Zhao S., Shi T., Zhang G. (2019). NF-kappaB signaling and integrin-beta1 inhibition attenuates osteosarcoma metastasis via increased cell apoptosis. Int. J. Biol. Macromol..

[10] Yang J., Zhang W. (2013). New molecular insights into osteosarcoma targeted therapy. Curr. Opin. Oncol..

[11] Hou C.H., Yang R.S., Tsao Y.T. (2018). Connective tissue growth factor stimulates osteosarcoma cell migration and induces osteosarcoma metastasis by upregulating VCAM-1 expression. Biochem. Pharmacol..

[12] Shi K., Wang S.L., Shen B., Yu F.Q., Weng D.F., Lin J.H. (2019). Clinicopathological and prognostic values of fibronectin and integrin αvβ3 expression in primary osteosarcoma. World J. Surg. Oncol..

[13] Lei Y., Junxin C., Yongcan H., Xiaoguang L., Binsheng Y. (2020). Role of microRNAs in the crosstalk between osteosarcoma cells and the tumour microenvironment. J. Bone Oncol..

[14] Cai W., Xu Y., Zuo W., Su Z. (2019). MicroR-542-3p can mediate ILK and further inhibit cell proliferation, migration and invasion in osteosarcoma cells. Aging.

[15] Wang D., Tang L., Wu H., Wang K., Gu D. (2018). MiR-127-3p inhibits cell growth and invasiveness by targeting ITGA6 in human osteosarcoma. IUBMB Life.

[16] Luo Z., Li D., Luo X., Li L., Gu S., Yu L., Ma Y. (2016). Decreased Expression of miR-548c-3p in Osteosarcoma Contributes to Cell Proliferation Via Targeting ITGAV. Cancer Biother. Radiopharm..

[17] Mukaida N., Sasaki S.I., Baba T. (2020). CCL4 Signaling in the Tumor Microenvironment. Adv. Exp. Med. Biol..

[18] Korbecki J., Grochans S., Gutowska I., Barczak K., Baranowska-Bosiacka I. (2020). CC Chemokines in a Tumor: A Review of Pro-Cancer and Anti-Cancer Properties of Receptors CCR5, CCR6, CCR7, CCR8, CCR9, and CCR10 Ligands. Int. J. Mol. Sci..

[19] Lee D., Shin K.J., Kim D.W., Yoon K.A., Choi Y.J., Lee B.N.R., Cho J.Y. (2018). CCL4 enhances preosteoclast migration and its receptor CCR5 downregulation by RANKL promotes osteoclastogenesis. Cell Death Dis..

[20] Sasaki S., Baba T., Nishimura T., Hayakawa Y., Hashimoto S., Gotoh N., Mukaida N. (2016). Essential roles of the interaction between cancer cell-derived chemokine, CCL4, and intra-bone CCR5-expressing fibroblasts in breast cancer bone metastasis. Cancer Lett..

[21] Lien M.Y., Tsai H.C., Chang A.C., Tsai M.H., Hua C.H., Wang S.W., Tang C.H. (2018). Chemokine CCL4 Induces Vascular Endothelial Growth Factor C Expression and Lymphangiogenesis by miR-195-3p in Oral Squamous Cell Carcinoma. Front. Immunol..

[22] Jiang K., Yao G., Hu L., Yan Y., Liu J., Shi J., Chang Y., Zhang Y., Liang D., Shen D. (2020). MOB2 suppresses GBM cell migration and invasion via regulation of FAK/Akt and cAMP/PKA signaling. Cell Death Dis..

[23] Wu Y.J., Lin S.H., Din Z.H., Su J.H., Liu C.I. (2019). Sinulariolide Inhibits Gastric Cancer Cell Migration and Invasion through Downregulation of the EMT Process and Suppression of FAK/PI3K/AKT/mTOR and MAPKs Signaling Pathways. Mar. Drugs.

[24] Luo J., Yao J.F., Deng X.F., Zheng X.D., Jia M., Wang Y.Q., Huang Y., Zhu J.H. (2018). 14, 15-EET induces breast cancer cell EMT and cisplatin resistance by up-regulating integrin alphavbeta3 and activating FAK/PI3K/AKT signaling. J. Exp. Clin. Cancer Res..

[25] Bhattarai D., Xu X., Lee K. (2018). Hypoxia-inducible factor-1 (HIF-1) inhibitors from the last decade (2007 to 2016): A "structure-activity relationship" perspective. Med. Res. Rev..

[26] Hayashi Y., Yokota A., Harada H., Huang G. (2019). Hypoxia/pseudohypoxia-mediated activation of hypoxia-inducible factor-1alpha in cancer. Cancer Sci..

[27] Vangelista L., Vento S. (2017). The Expanding Therapeutic Perspective of CCR5 Blockade. Front. Immunol..

[28] Aldinucci D., Borghese C., Casagrande N. (2020). The CCL5/CCR5 Axis in Cancer Progression. Cancers.

[29] Aldinucci D., Casagrande N. (2018). Inhibition of the CCL5/CCR5 Axis against the Progression of Gastric Cancer. Int. J. Mol. Sci..

[30] Haag G.M., Halama N., Springfeld C., Grün B., Apostolidis L., Zschaebitz S., Dietrich M., Berger A.-K., Weber T.F., Zoernig I. (2020). Combined PD-1 inhibition (Pembrolizumab) and CCR5 inhibition (Maraviroc) for the treatment of refractory microsatellite stable (MSS) metastatic colorectal cancer (mCRC): First results of the PICCASSO phase I trial. J. Clin. Oncol..

[31] Halama N., Zoernig I., Berthel A., Kahlert C., Klupp F., Suarez-Carmona M., Suetterlin T., Brand K., Krauss J., Lasitschka F. (2016). Tumoral Immune Cell Exploitation in Colorectal Cancer Metastases Can Be Targeted Effectively by Anti-CCR5 Therapy in Cancer Patients. Cancer Cell.

[32] Peng X., Gao H., Xu R., Wang H., Mei J., Liu C. (2020). The interplay between HIF-1α and noncoding RNAs in cancer. J. Exp. Clin. Cancer Res..

[33] Zhu S., He C., Deng S., Li X., Cui S., Zeng Z., Liu M., Zhao S., Chen J., Jin Y. (2016). MiR-548an, Transcriptionally Downregulated by HIF1α/HDAC1, Suppresses Tumorigenesis of Pancreatic Cancer by Targeting Vimentin Expression. Mol. Cancer Ther..

[34] Liu Z., Wang Y., Dou C., Xu M., Sun L., Wang L., Yao B., Li Q., Yang W., Tu K. (2018). Hypoxia-induced up-regulation of VASP promotes invasiveness and metastasis of hepatocellular carcinoma. Theranostics.

[35] Johanson T.M., Lew A.M., Chong M.M. (2013). MicroRNA-independent roles of the RNase III enzymes Drosha and Dicer. Open Biol..

[36] Shen J., Xia W., Khotskaya Y.B., Huo L., Nakanishi K., Lim S.O., Du Y., Wang Y., Chang W.C., Chen C.H. (2013). EGFR modulates microRNA maturation in response to hypoxia through phosphorylation of AGO2. Nature.

[37] Alday-Parejo B., Stupp R., Ruegg C. (2019). Are Integrins Still Practicable Targets for Anti-Cancer Therapy?. Cancers.

[38] Valdembri D., Serini G. (2021). The roles of integrins in cancer. Fac. Rev..

[39] Desgrosellier J.S., Cheresh D.A. (2010). Integrins in cancer: Biological implications and therapeutic opportunities. Nat. Rev. Cancer.

[40] Cheng T.M., Chang W.J., Chu H.Y., De Luca R., Pedersen J.Z., Incerpi S., Li Z.L., Shih Y.J., Lin H.Y., Wang K. (2021). Nano-Strategies Targeting the Integrin alphavbeta3 Network for Cancer Therapy. Cells.

[41] Ren P., Sun D., Xin D., Ma W., Chen P., Gao H., Zhang S., Gong M. (2014). Serum amyloid A promotes osteosarcoma invasion via upregulating alphavbeta3 integrin. Mol. Med. Rep..

[42] Stupp R., Hegi M.E., Gorlia T., Erridge S.C., Perry J., Hong Y.K., Aldape K.D., Lhermitte B., Pietsch T., Grujicic D. (2014). Cilengitide combined with standard treatment for patients with newly diagnosed glioblastoma with methylated MGMT promoter (CENTRIC EORTC 26071-22072 study): A multicentre, randomised, open-label, phase 3 trial. Lancet Oncol..

[43] Tsai H.C., Cheng S.P., Han C.K., Huang Y.L., Wang S.W., Lee J.J., Lai C.T., Fong Y.C., Tang C.H. (2019). Resistin enhances angiogenesis in osteosarcoma via the MAPK signaling pathway. Aging.

[44] Tsai H.C., Chang A.C., Tsai C.H., Huang Y.L., Gan L., Chen C.K., Liu S.C., Huang T.Y., Fong Y.C., Tang C.H. (2019). CCN2 promotes drug resistance in osteosarcoma by enhancing ABCG2 expression. J. Cell Physiol..

[45] Lee H.-P., Liu S.-C., Wang Y.-H., Chen B.-C., Chen H.-T., Li T.-M., Huang W.-C., Hsu C.-J., Wu Y.-C., Tang C.-H. (2021). Cordycerebroside A suppresses VCAM-dependent monocyte adhesion in osteoarthritis synovial fibroblasts by inhibiting MEK/ERK/AP-1 signaling. J. Funct. Foods.

[46] Lee H.-P., Chen P.-C., Wang S.-W., Fong Y.-C., Tsai C.-H., Tsai F.-J., Chung J.-G., Huang C.-Y., Yang J.-S., Hsu Y.-M. (2019). Plumbagin suppresses endothelial progenitor cell-related angiogenesis in vitro and in vivo. J. Funct. Foods.

[47] Tsai H.C., Huang C.Y., Su H.L., Tang C.H. (2014). CTGF increases drug resistance to paclitaxel by upregulating survivin expression in human osteosarcoma cells. Biochim. Biophys. Acta.

[48] Liu S.-C., Tsai C.-H., Wu T.-Y., Tsai C.-H., Tsai F.-J., Chung J.-G., Huang C.-Y., Yang J.-S., Hsu Y.-M., Yin M.-C. (2019). Soya-cerebroside reduces IL-1β-induced MMP-1 production in chondrocytes and inhibits cartilage degradation: Implications for the treatment of osteoarthritis. Food Agric. Immunol..

[49] Lee H.-P., Wu Y.-C., Chen B.-C., Liu S.-C., Li T.-M., Huang W.-C., Hsu C.-J., Tang C.-H. (2020). Soya-cerebroside reduces interleukin production in human rheumatoid arthritis synovial fibroblasts by inhibiting the ERK, NF-κB and AP-1 signalling pathways. Food Agric. Immunol..

[50] Lee H.-P., Wang S.-W., Wu Y.-C., Lin L.-W., Tsai F.-J., Yang J.-S., Li T.-M., Tang C.-H. (2020). Soya-cerebroside inhibits VEGF-facilitated angiogenesis in endothelial progenitor cells. Food Agric. Immunol..

