# Supplementary material for: CCL4 Stimulates Cell Migration in Human Osteosarcoma via the mir-3927-3p/Integrin αvβ3 Axis
Source: Int J Mol Sci. 2021 Nov 25;22(23):12737. doi: 10.3390/ijms222312737 (PMC8657600; doi:10.3390/ijms222312737)
Supplement: Supplementary file 1 [file ijms-22-12737-s001.zip › ijms-1460515-supplementary.pdf]

Table S1. Details about the antibodies used in the study.

Name of antibody	*Dilution for WB*	*Dilution for IHC*	*Dilution for flow cytometry*	*Company*	*Product number*
CCL4	—	1:200	—	Santa Cruz Biotechnology	sc-130330
Integrin αv/β3	—	1:200	1:200	Santa Cruz Biotechnology	sc-7312
p-FAK	1:1000	—	—	Cell Signaling Technology	3283S
FAK	1:1000	—	—	Santa Cruz Biotechnology	sc-271195
p-AKT	1:1000	—	—	Cell Signaling Technology	4060S
AKT	1:1000	—	—	Santa Cruz Biotechnology	sc-5298
HIF-1α	1:1000	—	—	Abcam	ab1
α-Tubulin	1:5000	—	—	Santa Cruz Biotechnology	sc-5286

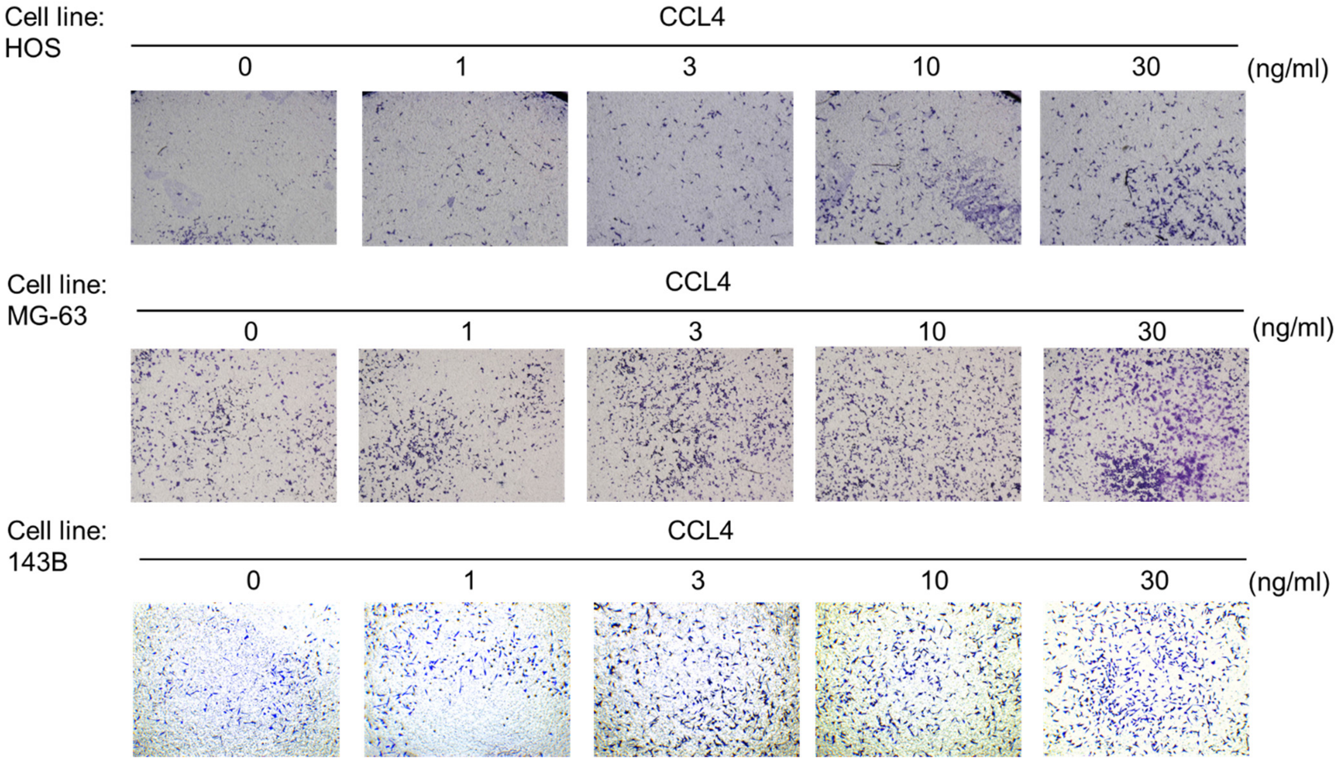

Figure S1. **Raw data from the cell migration experiments.** Cells were incubated with different concentrations of CCL4 (0–30 ng/ml) for 24 h, then cell migration ability was examined by the Transwell assay.

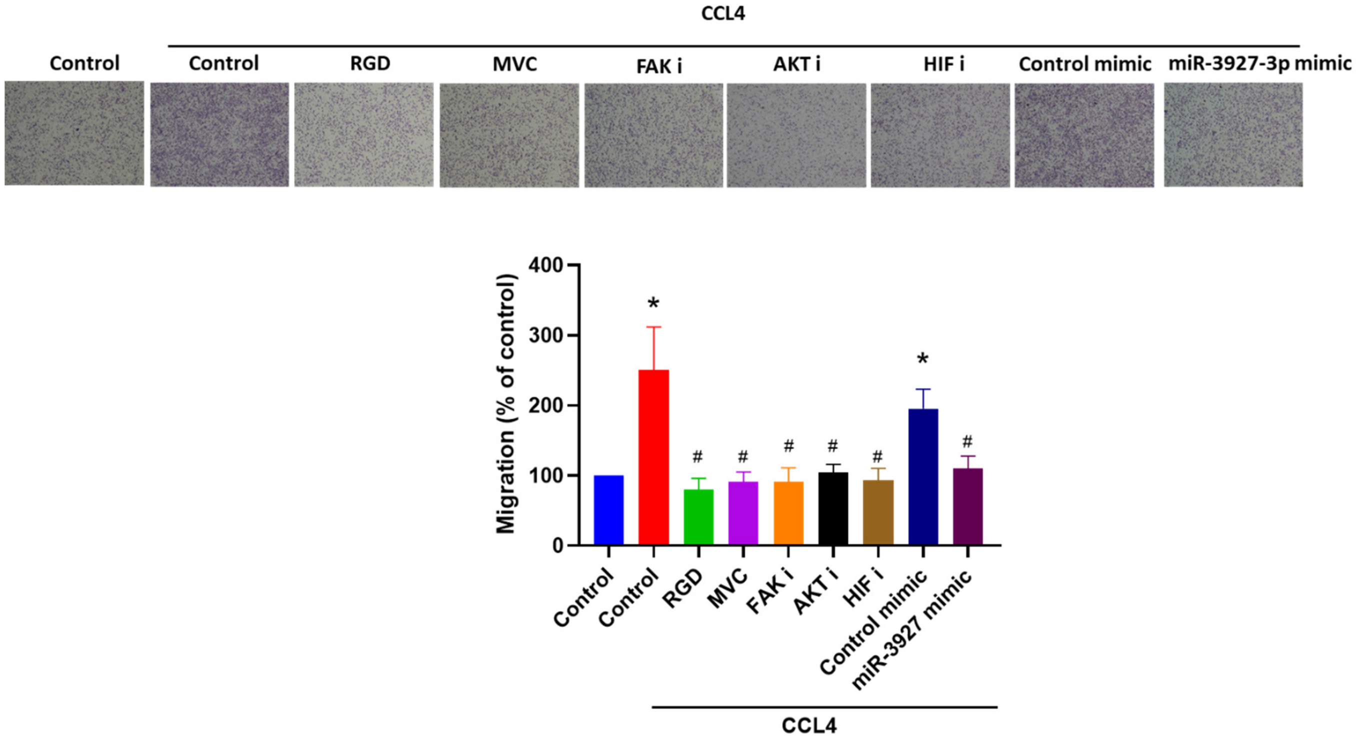

Figure S2. **CCL4-induced osteosarcoma cell migration via CCR5, the FAK/AKT/HIF-1α signaling pathway, miR-3927-3p and integrin αvβ3.** MG-63 cells were treated with RGD (100 nM), MVC (5 uM), FAK i (10 uM), AKT i (10 uM), or HIF i (10 uM) for 30 min, or transfected with control or miR-3927-3p mimic, then stimulated with CCL4 for 24 h. Levels of cell migratory abilities were examined by the Transwell assay. The results were obtained from three independent experiments and are expressed as the means ± SD. * $p < 0.05$ compared with controls; # $p < 0.05$ compared with CCL4-treated controls.

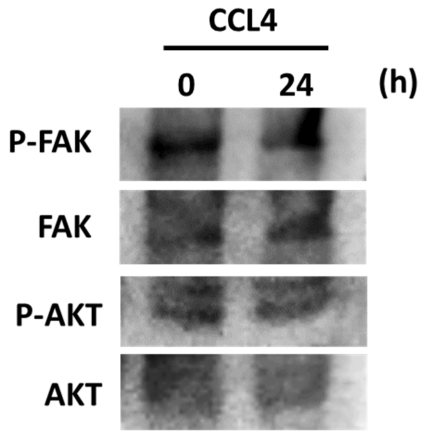

Figure S3. **Levels of p-FAK and p-AKT expression after 24 h of CCL4 exposure.** 143B cells were incubated with CCL4 (30 ng/ml) for 24 h, then FAK and AKT activation was examined by the Western blot.

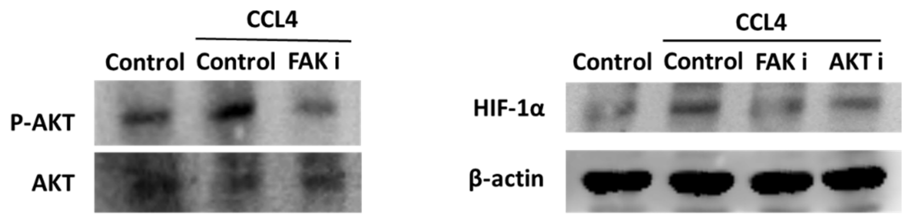

Figure S4. **Levels of p-AKT and HIF-1α expression after treatment with FAK and AKT inhibitors.** 143B cells were treated with FAK i (10 uM) or AKT i (10 uM) for 30 min, then with CCL4 (30 ng/ml) for another 30 min. AKT and HIF-1α activation was examined by Western blot.
